# Round Well Inset for Uniform Electric Field Distribution in Electroporation Applications

**DOI:** 10.3390/bioengineering12020203

**Published:** 2025-02-19

**Authors:** Praveen Sahu, Marco Barozzi, Paolo Di Barba, Maria Evelina Mognaschi, Monica La Mura, Patrizia Lamberti, Michele Forzan, Maria Teresa Conconi, Ignacio Camarillo, Raji Sundararajan, Elisabetta Sieni

**Affiliations:** 1 School of Engineering Technology, Purdue University, West Lafayette, IN 47907, USA; psahu@purdue.edu; 2 Science and High Technology Department, University of Insubria, 22100 Como, Italy; marco.barozzi@uninsubria.it; 3 Department of Industrial and Information Engineering, University of Pavia, 27100 Pavia, Italy; paolo.dibarba@unipv.it (P.D.B.); eve.mognaschi@unipv.it (M.E.M.); 4 Department of Information and Electrical Engineering and Applied Mathematics, University of Salerno, 84084 Fisciano, Italy; mlamura@unisa.it (M.L.M.); plamberti@unisa.it (P.L.); 5 Department of Industrial Engineering, University of Padova, 35122 Padova, Italy; michele.forzan@unipd.it; 6 Department of Pharmaceutical and Pharmacological Sciences, University of Padova, 35122 Padova, Italy; mariateresa.conconi@unipd.it; 7 Department of Biological Sciences, Purdue University, West Lafayette, IN 47907, USA; ignacio@purdue.edu; 8 Department of Theoretical and Applied Sciences, University of Insubria, 21100 Varese, Italy

**Keywords:** electroporation, electric field distribution, inset, 3D printing, finite element analysis

## Abstract

Uniform electric field distribution in electroporation is crucial for achieving the effective localized delivery of drug molecules. Currently, in vitro electroporation studies on adherent cells lack a standardized test setup for obtaining consistent and repeatable results, unlike in vitro electroporation studies on cell suspensions that use electroporation cuvettes, which provide uniform electric field distribution. Considering this, we designed, built, and tested a novel inset design for low-volume round well plates, such as the 24- and 96-well plates which are most commonly used in cell culture labs. The inset design was realized using 3D printing and experimentally tested using potato phantoms and HeLa cells. Finite element analysis (FEA) was used to compute the electric field distribution in the round well plates with and without the inset. The FEA indicated that the electric field contour map at the bottom of the well with the inset had a more uniform electric field distribution, with an average value close to the expected 1000 V/cm. In contrast, it was only 840 V/cm without the inset, indicating non-uniform electric field distribution. Uniform electric fields were also obtained using the inset for the potato phantoms and the HeLa cells, indicating the merit of the inset and its usability with low-volume cell culture well plates, which enable the transfer of cells for various assays without additional steps, as well as its cost-effectiveness.

## 1. Introduction

Electroporation (EP) uses voltage pulses to permeabilize cell membranes. Cell membrane electroporation is used in clinical treatment to improve drug uptake (reversible electroporation) or to destroy cells (irreversible electroporation) [1,2,3,4]. In Europe, reversible electroporation is a well-established clinical therapy, whereas irreversible electroporation is used in the USA to treat locally advanced, inoperable pancreatic and other solid cancers [5,6,7,8].

Electroporation efficiency depends on the distribution of the electric field within the target tissue [9]. Equation (1) gives the local electric field vector ***E*** (Vm^−1^) in a homogenous biological tissue [10].

(1) $$\frac{\partial }{\partial x}\left(\sigma_{x}\left(E\right)\cdot \frac{\partial U}{\partial x}\right)+\frac{\partial }{\partial y}\left(\sigma_{y}\left(E\right)\cdot \frac{\partial U}{\partial y}\right)=0$$

where *x* and *y* are the direction vectors, σ is the electrical conductivity (Sm^−1^) of an anisotropic medium with σ*_x_* and σ*_y_* as the electrical conductivity tensorial components that are function of *E*, *U* (V) is the electric potential with *E* =−∇U. However, biological tissues are not homogenous, and cells are immersed in an extracellular matrix (ECM) composed of collagen and other ECM components with different conductivities. It results in a non-homogenous conductive environment and modifies the electric field distribution, as demonstrated in [11].

A homogeneous electric field distribution is necessary for electroporation, as it ensures that all cells are exposed to the same electric field strength, leading to more consistent and predictable results. With heterogeneous electric field distribution, some cells would be exposed to a higher electric field strength than others. This can result in the over-electroporation of some cells and the under-electroporation of others, which can reduce the overall effectiveness of the treatment. Several studies have demonstrated the importance of uniform electric field distribution in electroporation. For example, Miklavčič et al. [12] showed the use of different electrode sets to obtain uniform electric field distribution and a more effective antitumor response in a mouse model of electrochemotherapy. Ivorra et al. [13] used conductive gels to homogenize the electric field distribution to improve the antitumor efficacy of electrochemotherapy in sarcoma tumors implanted in mice. Thus, external accessories are needed to obtain uniform electric field distribution in adherent cell culture studies with round wells. These prior studies demonstrated that homogenizing the electric field distribution is vital for improving electroporation-based treatments. However, the in vitro preclinical electroporation studies on tissue or cell culture lack a standardized testing setup for achieving highly consistent and repeatable results. The study by A. Briko et al. [14] used electroporation for tumors. Here, the numerical simulations modeled electric field distribution for efficient electroporation procedures, emphasizing the optimal electrode configurations for maximum tumor coverage. Overlapping fields were necessary for tumors over 10 mm. The study suggested a planning methodology based on the physical parameters, with limitations including the need for 3D reconstruction and experimental validation for irregularly shaped tumors.

The homogeneity and uniformity of the electric field in a round well typical of a standard culture plate is not guaranteed with the use of plate electrodes, since the electric field is distributed not only throughout the bottom of the entire culture chamber where there is a conductive medium but also adjacent to where the electrode is facing [15,16,17]. In particular, a not-uniform field in proximity of the electrode edge is generated, where some cells can experience an electric field intensity that is lower than the level in the area between the electrodes [16,18].

**Figure 1:**
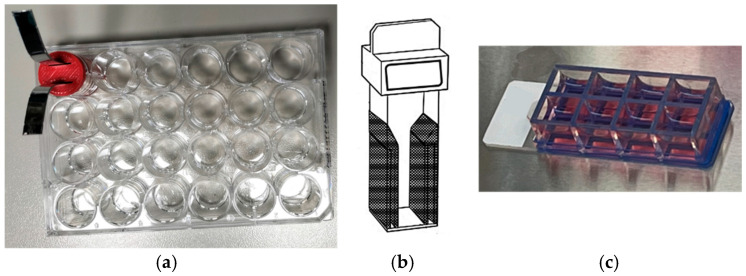
(**a**) 24-well plates with the proposed inset, (**b**) cell suspension cuvette, and (**c**) Chamber Slides^TM^ System.

Considering this, in this study, we designed, built, and tested a novel inset design that homogenizes the electric field distribution in low-volume round well plates, such as 24- and 96-well plates (Figure 1a), which are most commonly used in cell culture studies, leading to electrical conditions similar to those of a cell suspension in an electroporation cuvette (Figure 1b) or a Chamber Slides^TM^ System (Figure 1c). We designed an inset for round wells to create a regular rectangular area well fitted by the plate electrodes. We built it using 3D printing and tested it on biological tissues, such as potato phantoms [19,20] and HeLa cells. We also used finite element analysis (FEA) to model and simulate the electric field distribution in low-volume round wells with and without the inset.

## 2. Materials and Methods

### 2.1. Inset Conceptualization

**Figure 2:**
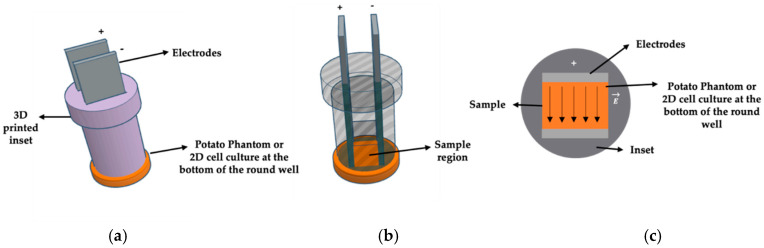
Illustration of the inset concept to obtain uniform electric field distribution (Not to scale). (**a**) Parallel plate electrodes, with a 3D-printed inset, designed for a round well, inserted into a potato phantom sample at the bottom of the round well. (**b**) Transparent 3D view showing the sample region. (**c**) A cross-sectional view at the bottom of the well through the potato sample, with indicated electric field distribution and insulated region by the non-conducting inset.

Figure 2 shows the proposed inset design, facilitating the electroporation of the potato phantom samples and the HeLa cells in round well plates. The design consists of a slot for parallel plate electrodes and a cylindrical structure specifically engineered to fit within a round well. The inset slot maintains a consistent distance between the electrodes every time electroporation is performed. Figure 2a shows the parallel plate electrodes through a 3D-printed inset, designed for a round well, inserted into a potato phantom sample at the bottom of the round well. Figure 2b illustrates the transparent 3D view showing the sample region. The bottom of the well inset creates a region for a uniform localized electrical field, as shown in Figure 1c. It enables precise and controlled investigations.

### 2.2. Finite Element Analysis (FEA)

**Figure 3:**
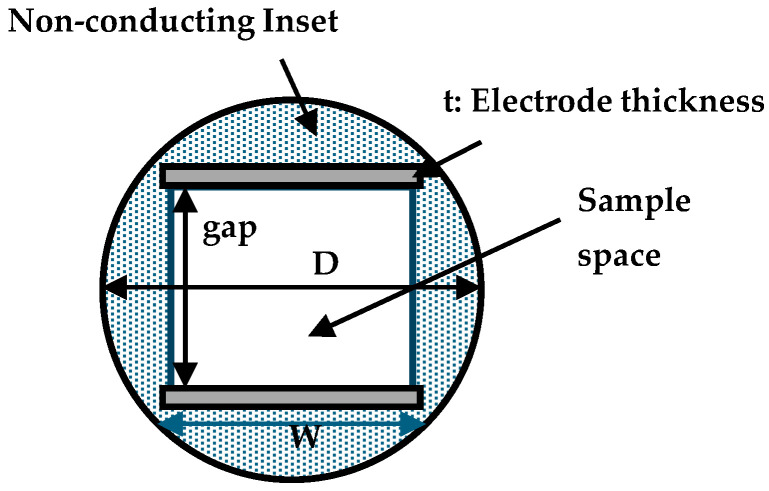
Cross-section of the FEA mesh for the designed well inset.

The FEA model is a 3D geometry, representing the round well with an internal diameter of D = 15 mm and a height of 20 mm for the 24-well round plate and D = 7 mm, with a height of 12 mm for the 96-well round plate. Figure 3 shows a cross-section, and Table 1 shows the dimensions.

The electrodes were modeled as stainless-steel plates with thicknesses of 1 mm (plate width 10 mm) or 0.2 mm (plate width 3.3 mm) for the 24- or the 96-well plates, respectively. The plate gap was 7 mm or 2.9 mm for the 24- or the 96-well plates. The dotted area represents the inset, made of 100% biodegradable, non-toxic, and odorless polylactic acid (PLA) material. The gray rectangles represent the sections of the electrodes. FEA analyses were performed with and without the inset. In both cases, the electrode position was maintained at the same location for comparative study.

The plate electrodes were energized, applying a voltage difference *U* of 700 V or 290 V to the 24- or the 96-well plates, respectively, to obtain a uniform electric field strength of 1000 V/cm. In the model, the PLA resin of the inset is a non-conducting material, while the sample space at the bottom of the well has a conductivity of 0.2 S/m.

The FEA was performed as a conduction problem, using Altair Flux 3D software (Altair Engineering, Troy, MI, USA, [21]), To estimate the electric field distribution in the well volume, Laplace’s equation of scalar electric potential [22] is considered:

∇⋅σ(∇U) = 0(2)

where σ is the electrical conductivity of the material. By imposing the Dirichlet conditions [23] at the electrodes’ surfaces, the effect of the application of a 100 μs long rectangular voltage pulse is studied as a stationary case by fixing one electrode potential at 0 V, and the other one at the maximum pulse value, whereas the Neumann boundary condition was applied [24] for the remaining external sides The mesh of the FEA model had 105,700 nodes and 611,300 elements for the 24-well plate and 8000 nodes and 43,800 elements for the 96-well plate. The electric field strength was studied in two normal planes, XZ and YZ.

### 2.3. Inset Realization Using 3D Printing

**Figure 4:**
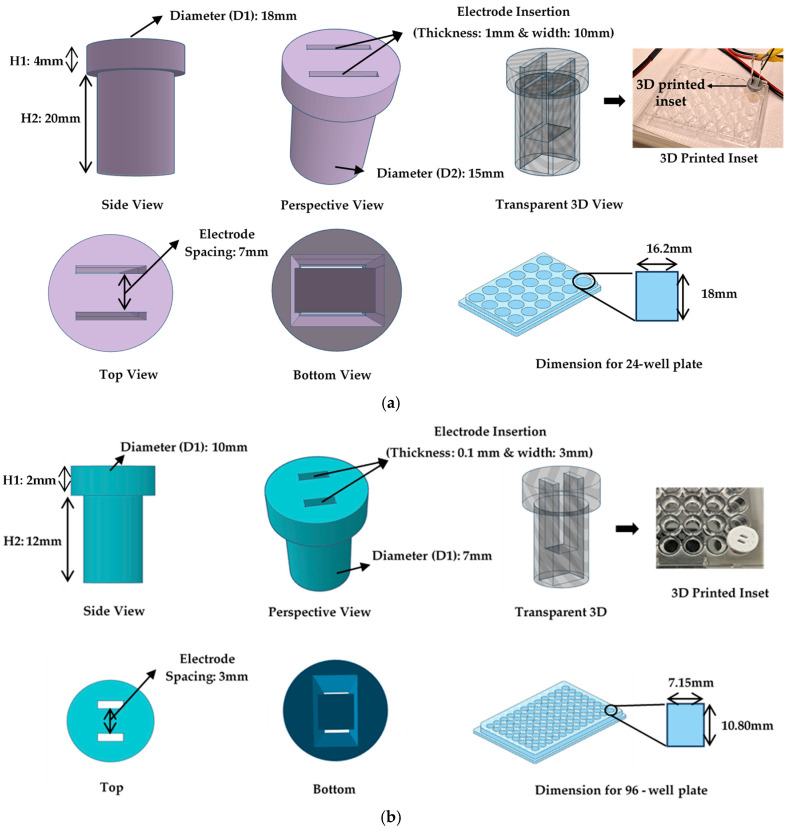
3D—CAD inset design showing the side view, perspective, transparent 3D view, top view, bottom view, and the 96-well plates along with actual 3D-printed part for: (**a**) 24-well plate inset; (**b**) 96-well plate inset (all the dimensions for inset are mentioned in Table 2).

After the FEA analysis, the inset for the 24-well plate was built using additive manufacturing (Figure 4).

The inset was designed using the Thinker Computer-Aided Design (CAD) software (a free web app for 3D design developed under Autodesk Free Products License, Release 2024), ensuring they fit the plates precisely. A suitable biocompatibility PLA filament was selected for 3D printing [25]. The CAD design (Figure 4a) was then converted into a format suitable for the 3D printer (STL), and the printing parameters were configured following the manufacturer’s guidelines (Ultimaker [26]). The inset was meticulously constructed by the 3D printer, layer by layer. After printing, care was taken to remove any imperfections, and the fit was verified by testing the inset on the plates, ensuring that the electrodes aligned parallelly. Before the experiment, the insets were sterilized using 70% ethanol and 1 h of UV exposure under a biological hood. These 3D-printed cylindrical insets served as precise guides for the electroporation electrodes, enhancing the accuracy and consistency of electroporation experiments on multi-well plates and eliminating round borders, where the electric field has a lower strength with respect to the area between plates. Figure 4b shows the same for the 96-well plate. All the dimensions for the inset are mentioned in Table 2.

**Table 2:** Dimensions of the 24- and 96-well plates’ with inset supplementation Figure 4a and Figure 4b.

SN	Parameter	24—Well Plate	96—Well Plate
1	Height (H1)	4 mm	2 mm
2	Height (H2)	20 mm	12 mm
3	Diameter (D1)	18 mm	10 mm
4	Diameter (D2)	15 mm	7 mm
5	Electrode space	7 mm	3 mm
6	Thickness	1 mm	0.1 mm
7	Width	10 mm	3 mm

### 2.4. Potato Phantom Preparation

**Figure 5:**
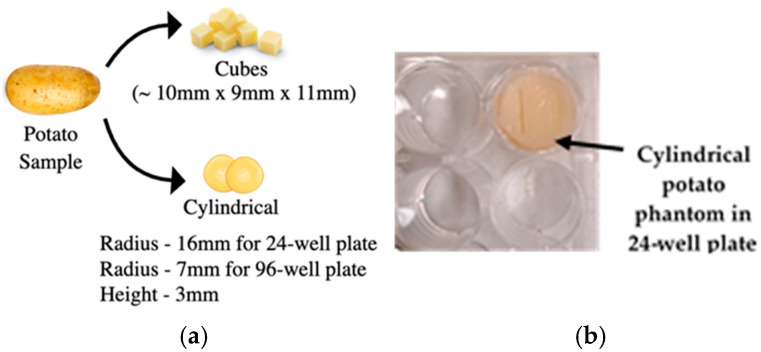
Potato sample: (**a**) Cut into cubes and cylindrical shape; (**b**) Cylindrical potato phantom sample inside a 24-well plate.

Fresh potatoes of uniform size were selected for the study. These potatoes were bought from the local market and were thoroughly washed and cleaned to remove any surface impurities. Using a knife and cutting board, the potatoes were meticulously cut into cylindrical shapes measuring 16.5 mm in diameter and 3 mm in height for the 24-well round plate and 10 mm × 9 mm × 11 mm for a rectangular well chamber (Figure 5a). Figure 5b shows a cylindrical potato in a 24-well plate.

Special attention was given to ensure that the potato phantoms had flat ends, which was crucial for achieving consistent electroporation results. The prepared potato phantoms were immediately electroporated and then stored at room temperature. After 24 h, a picture of the sample surface was captured for further analyses.

### 2.5. Potato Phantom Experiments

**Figure 6:**
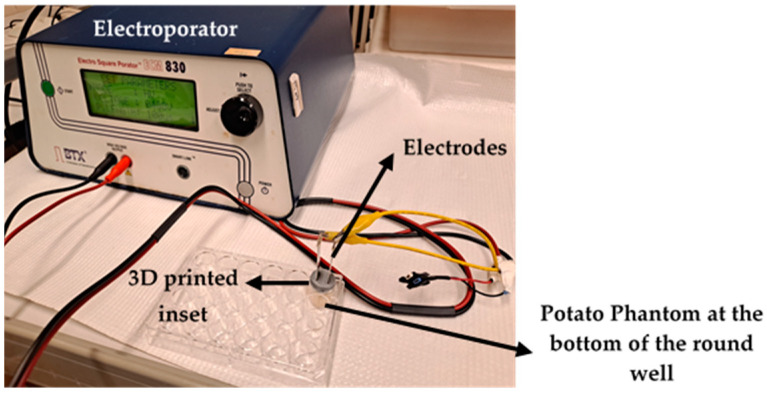
An experimental setup consisting of an electroporator and a 3D-printed inset inside a 24-well plate with stainless steel parallel with cylindrical potato phantom.

The experimental setup includes potato samples cut to fit 24 or 96-well plates with the inset. The stainless-steel plate electrodes are inserted, as shown in Figure 5b. The potato samples are electroporated using EPS01 or the BTX-ECM830 voltage pulse generator (Figure 6) following the electroporation protocol.

### 2.6. Electroporation Protocol

The electroporation voltage pulses were applied using the EPS01 voltage pulse generator (Igea SpA, Carpi (MO), Italy [27]) or the BTX-ECM830 voltage pulse generator (BTX, Holliston, MA, USA). A voltage difference of 700 V or 290 V was used to obtain a uniform field of 1000 V/cm. The voltage pulses were applied using stainless steel electrodes with two parallel plates of 30 mm long and a gap of 7 mm.

### 2.7. Cell Culture Studies

HELA cells (cervical cancer line), purchased from ATCC (https://www.atcc.org (accessed on 15 September 2023)), were cultured in DMEM high glucose, supplemented with 1% penicillin/streptomycin, 1% L-glutamine, and 10% FBS at 37 °C in a humified atmosphere with 5% CO_2_ (SteriCult CO_2_ incubator, Thermo Electron Corporation, Waltham, MA, USA). Cells (50 × 10^3^) were seeded in 24-well plates (internal diameter 15 mm) or in an 8-well chamber slide (section 10 × 9 mm) with 500 μL of cell medium.

Before electroporation, the medium was substituted with 300 μL of electroporation buffer (10 mM K_2_HPO_4_/KH_2_PO_4_, 1 mM MgCl_2_, and 250 mM sucrose, pH 7.4 [28]) with 8 μL of propidium iodide (PI) solution (1 mg/1 mL). Eight voltage pulses were applied using a plate electrode (side was 1 cm long with a gap of 7 mm). After electroporation, the electroporation buffer was substituted with 300 μL of fresh medium and 5 μL of Hoechst 33342 (HOE) dye (1 mg/1 mL, Thermofisher, Waltham, MA, USA). While PI (red color) indicates cell electroporation, HOE (blue color) stains all the cells.

After electroporation, the cell cultures were observed with an inverted microscope Leica DI4000 (objective 20 × 0.35 DRY, camera DFC300FXR2-078921405). Both PI (excitation 538 nm, emission around 619 nm) and HOE (excitation 352 nm, emission around 455 nm) were visualized. For each culture, all the wells were scanned at 2.5× or 5× magnification, and red and blue images were recorded. The blue and red images were superposed using the software, LAS AF Lite (4.0, Leica, Deer Park, IL, USA). All the recorded images were composed in order to reconstruct the well bottom.

### 2.8. Image Analysis

The red levels of the intensity, measured in the range of 0–255, were analyzed using ImageJ program (1.50d, National Institutes of Health, USA) in cell culture images with fluorescence. The background intensity was determined by analyzing the color of areas without cells. The red intensity was sampled along the parallel lines and in the rectangle. The average intensity and median values were evaluated in the rectangles with the same number of pixels positioned in different subareas on the image.

## 3. Results and Discussion

### 3.1. FEA Simulation: Electric Field Strength Distributions Along XZ

**Figure 7:**
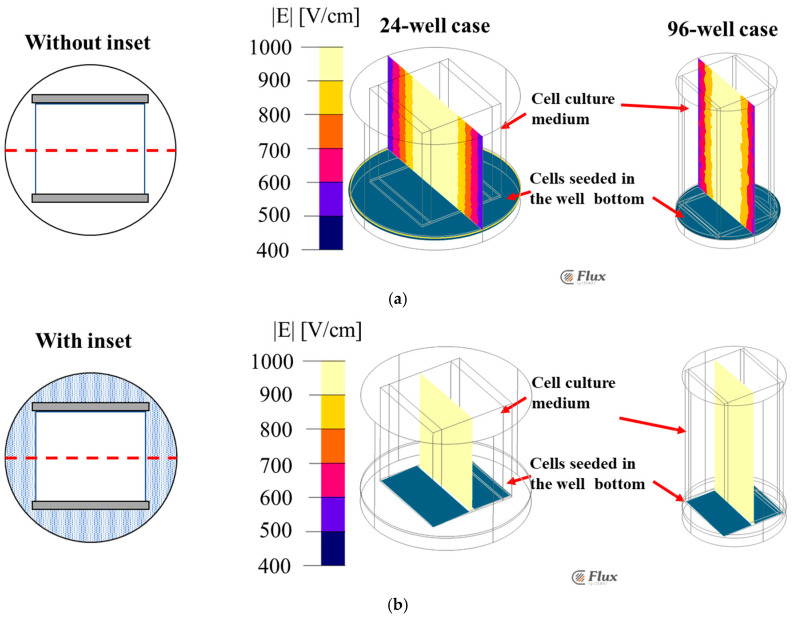
Electric field distribution contour along XZ plane for 24- and 96-well plate (**a**) without inset and (**b**) with inset. The color-coded legend shows the electric field value is V/m.

Figure 7 shows the XZ plane electric field strength distributions of the 24- and 96-well plates using the FEA with and without the inset.

Without the inset, the electric field intensity is 1050 V/cm (average value) in both the wells at the center. However, it varies from 900 V/cm to 500 V/cm at the edges, indicating the non-uniformity of the electric field distribution (Figure 7a). This aspect will lead to a non-uniform treatment of the total sample and is not a reliable evaluation of the electroporation. Thus, a properly designed inset could be a solution to improve the application of an electric field with the same homogeneity in all the parts of the treated sample. Figure 7b shows the uniform electric field distributions of these wells, with the proposed inset design, at an average intensity of 1050 V/cm throughout the volume.

### 3.2. FEA Simulation: Electric Field Strength Distributions Along YZ

**Figure 8:**
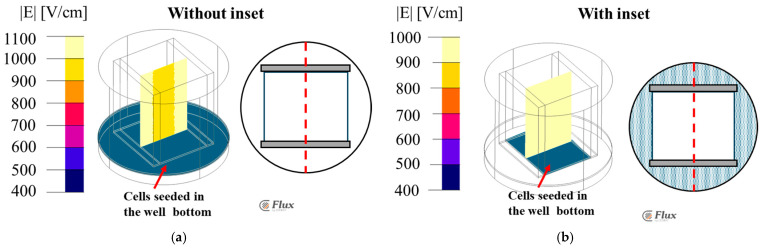
Electric field distribution contour along YZ for 24-well plate (**a**) without inset (**b**) with inset. The color-coded legend shows the electric field value is V/m.

Figure 8 shows the YZ plane electric field distribution in a 24-well plate, with the cells on the bottom with the media. Both with and without the inset were simulated. Without the inset, the electric field distribution is non-uniform, ranging from 900 V/cm to 1100 V/cm (Figure 8a). With the inset, the electric field is homogeneous at an intensity of 1050 V/cm (average) (Figure 8b). Thus, using the inset will be advantageous when the electric field homogeneity is mandatory, as in the case of an in vitro study for determining the electroporation threshold.

### 3.3. FEA Simulation: Potatophantom Electric Field Strength Distributions Along XZ

**Figure 9:**
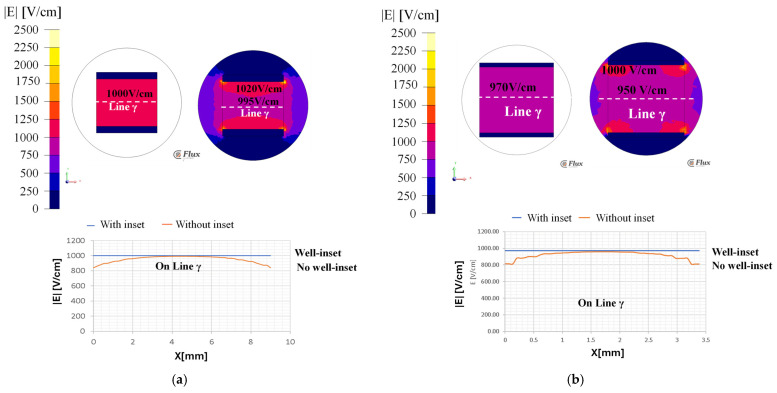
Electric field distribution contour along XZ for (**a**) 24-well plate and (**b**) 96-well plate. The color-coded legend shows the electric field value is V/m.

Figure 9 shows the FEA simulated electric field maps using the potato phantom, along the XZ plane distribution, for both with and without the inset for the 24-well plate (Figure 9a) and the 96-well plate (Figure 9b). Figure 9a shows the uniform electric field distribution of an intensity of 1000 V/cm along the line γ with the inset in a 24-well plate. However, without the inset, the inhomogeneous electric field distribution with field values ranges from 995 to 1020 V/cm, compared to 1000 V/cm as was designed. Figure 9b shows the electric field sampled along the line γ of 970 V/cm for a 96-well plate. The electric field strength at the electrode edge varies from 840 V/cm instead of 1000 V/cm in both the 24- and 96-well plates without the inset.

### 3.4. HeLa Cells Experiments

The case of the 24-well plate was verified experimentally using HeLa cells in adhesion and compared with a cell culture in a chamber slide. Both the cell cultures were electroporated using the same electrode and electric field intensity, and the cells were stained with blue and red dyes.

**Figure 10:**
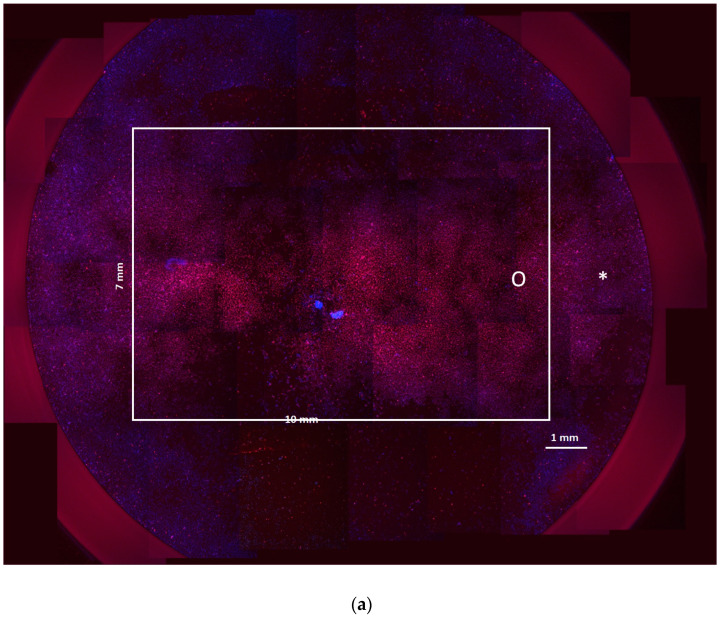

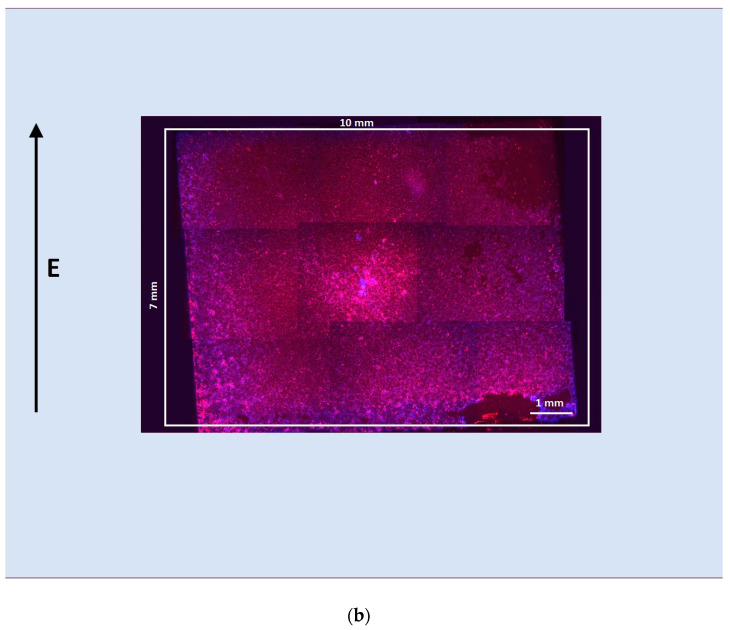
Fluorescence images of cell cultures after electroporation in (**a**) round well in a 24-well plate and (**b**) in a well of a chamber slide. The white star marks an area outside the electrode area but electroporated (red cells).

Figure 10 shows the electroporation of cells in adhesion cultured in a 24-well round plate and in a chamber slide. Since blue dye marks all the cells, whereas red dye only marks electroporated cells, the red and blue cells are a measure of cells electroporated and not electroporated. A different shade of red intensity means that electroporation of the cell membrane occurs with a difference in the number of pores [29,30,31]. Figure 10a represents the round well in which the electrode is centered and does not cover all the well area. Here, the white rectangle represents the area where the electrode is positioned in the cell culture. It can be seen that the areas outside the white square are affected by the electric field and are electroporated but at a different intensity, as evidenced by the lighter shade of red. Figure 10b shows what occurs in a rectangular well of a chamber slide, where the electrode fits the well shape. In this case, the electric field covers the entire chamber bottom, and the cells are all red.

**Figure 11:**
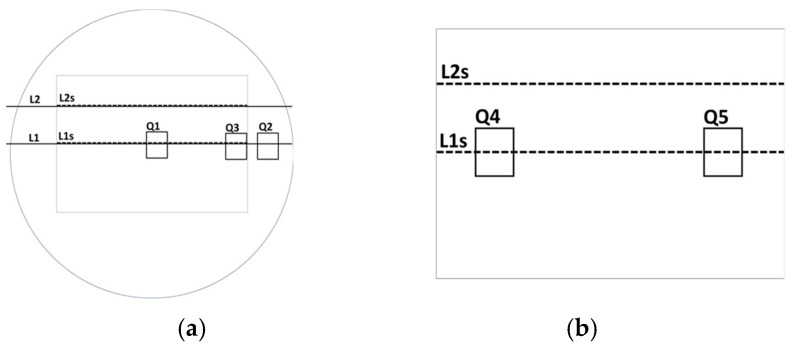
Sampling areas in the cell images for the analysis. (**a**) round well in a 24-well plate and (**b**) in a well of a chamber slide.

In Figure 10a, the white star marks an area outside the electrode area but electroporated (red cells), even if the red intensity is lower with respect to the one that occurs inside the white rectangle. The white circle marks the area in proximity to the rectangle edge, where the electric field in the simulation shows inhomogeneity. Figure 11 shows the lines and points, where the red color of the images was analyzed for the round well case (Figure 11a) and the chamber slide case (Figure 11b).

**Figure 12:**
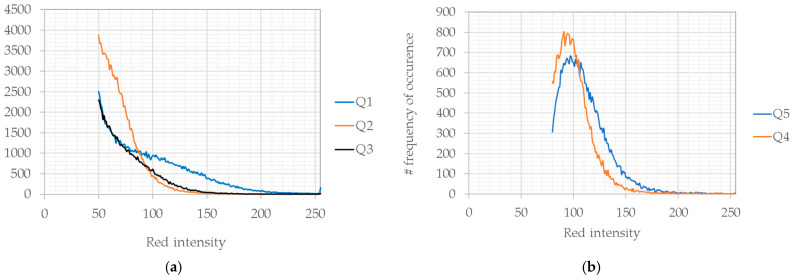
The frequency of red intensity on the analyzed rectangles for Figure 10. (**a**) a round well in a 24-well plate and (**b**) a well in a chamber slide.

Figure 12 indicates the analysis of the red intensity observed inside the rectangles in Figure 11. In each image, the area of rectangles is the same. The distribution of the red levels depends on the position of the rectangle. For each experiment, the background level was evaluated in areas without cells, as reported in Table 2.

Table 3 reports the average and median values obtained along lines L1s and L2s and in the squares Q1–Q5. From Figure 12, it is evident that in a round well, the electroporation also occurs in areas outside the electrode shape and with decreasing intensity along the border. Comparing these results with simulations in the same boundary area, the electric field has a lower intensity with respect to the center. Then, cells receiving a lower electric field intensity are consequently less affected by electroporation. However, in the rectangular chamber slide, all the cells were electroporated with similar intensity, as revealed by the analysis of the red intensity along the lines, L1s 2 L2s, and in the squares, Q4 and Q5.

These results support the simulation and demonstrate the necessity of a device that can adapt the electroporation area to the plate electrode.

### 3.5. Potato Phantoms Experiment

**Figure 13:**
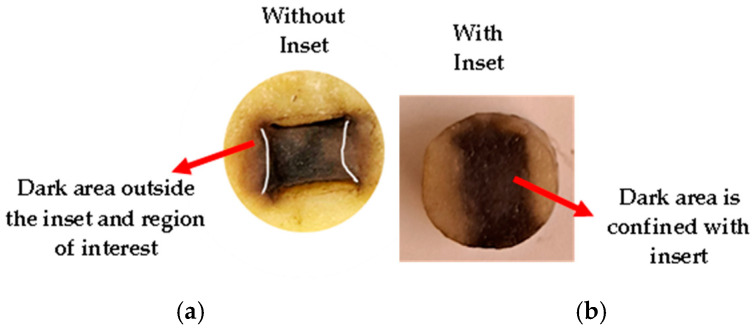
Potato phantom after 24 h treatment with 1000 V/cm (**a**) without inset, (**b**) with inset.

Figure 13 shows the electric field distribution in the potato phantoms, with and without the inset. Here, the brown (dark) area illustrates the electroporated region. Without the inset (Figure 13a), the electric field dispersed out of the volume delimited by the electrodes. This correlates well with the FEA simulation results. The brown region extends out of the region of interest, which might be problematic for in vitro and in vivo experiments for which a localized uniform electric field is desired in the cell sample, as a variable electric field intensity might affect the effect of electroporation treatment on the patients.

With the inset (Figure 13b), the electric field distribution was confined within the electroporated region—the brown color area. The electric field distribution correlated well with the FE simulation when 1000 V/cm was applied. The brown color is more intense in the center of the sample, and it is confined within the inset region and in the area of interest.

### 3.6. Realized Devices

**Figure 14:**
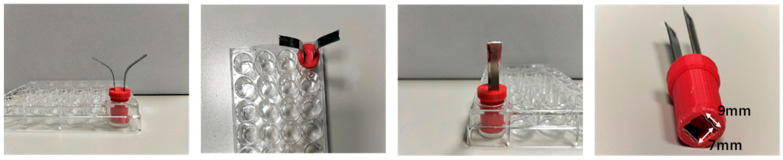
The realized device for 24-well plates (Up, bottom, and lateral faces).

**Figure 15:**
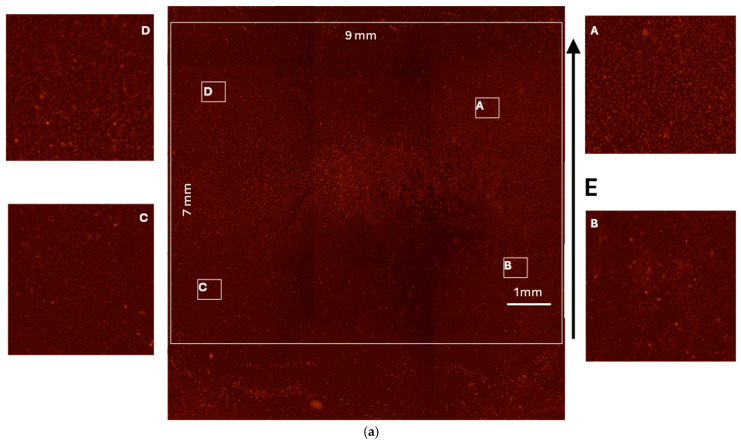

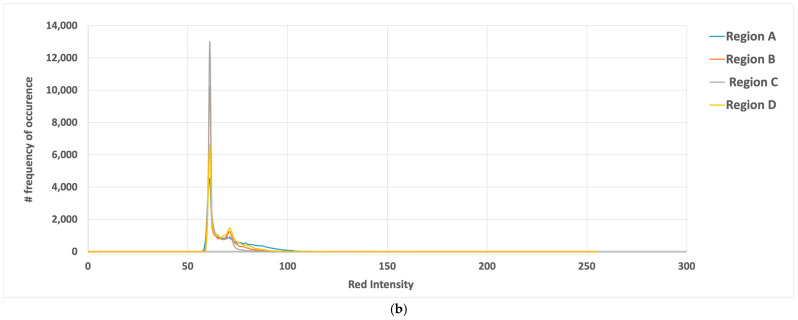
Electroporation results. (**a**) Using the inset in a 24-well plate. (**b**) Quantitative analysis: the frequency of red intensity on the analyzed rectangular regions—A, B, C, and D.

Figure 14 illustrates the realized device, sized for the 24 well plates (the up, bottom, and lateral faces), while Figure 15a shows the electroporation obtained in a 24-well plate using the inset, and Figure 15b illustrates the quantitative analysis. Lateral panels were magnified in the center imager to show the electroporation efficacy.

The quantitative analysis of electroporation efficacy, as shown in Figure 15b was conducted using fluorescence microscopy and intensity distribution measurements across a sample area of 9 mm × 7 mm. The fluorescence intensity distributions were examined in four distinct regions (A–D) of the electroporated sample, revealing remarkably consistent patterns. The intensity profiles showed a prominent peak at approximately 60 arbitrary units (a.u.) of red intensity, with Region C displaying the highest occurrence frequency at around 13,000 counts. A secondary peak was noted near 75 a.u., followed by a gradual decline in signal frequency beyond 100 a.u.

The spatial distribution of fluorescence, visualized through high-resolution microscopy, exhibited uniform patterns across all sampled regions, with individual fluorescent spots clearly distinguishable from the background. The microscopy data, accompanied by a 1 mm scale bar reference, demonstrated comparable fluorescence patterns in the magnified views of regions A through D, indicating homogeneous electroporation efficiency throughout the sample.

The consistency of the intensity distributions and spatial patterns across different regions suggests that the electroporation conditions were highly reproducible, with the majority of cells exhibiting similar levels of fluorescent marker uptake. This uniformity in both the quantitative intensity profiles and qualitative microscopy observations confirms the reliability and spatial consistency of the electroporation protocol.

## 4. Discussion

**Figure 16:**
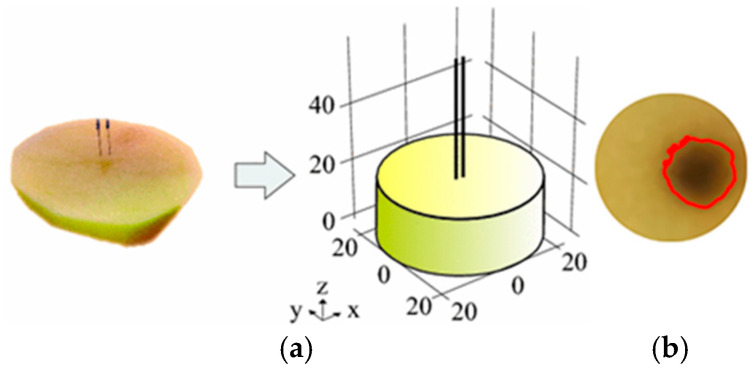
Use of potato phantom for electroporation studies by Berkenbrock et al. [34] (**a**) Potato phantom sample; (**b**) the red contour line shows the brown areas on potatoes phantom after 24 h of electroporation treatment using eight pulses of 100 µs at 1 Hz.

The use of potato phantoms as representative models for in vitro cell culture studies or in vivo studies is common, as has been undertaken by various researchers [15,16,17,18,20,32,33,34,35]. Figure 16 shows the results obtained by Berkenbrock et al. [34] using a potato phantom. They also obtained a brown region in the electroporated area.

The use of potato phantoms is consistent with the notion of the 3Rs in animal testing, i.e., reduction in the number of animals used, refinement of procedures to reduce distress, and the replacement of animals with non-animal techniques [33,34,36]. This is possible because potato phantoms have a similar texture and electrical conductivity to biological tissues. They are readily available, easy to prepare, inexpensive, and are safer than working with live animal subjects. In addition, their cellular structure provides a simplified analog for preliminary investigations and exhibits relatively consistent electrical properties within the same potato, contributing to the reproducibility of electroporation outcomes in a single experiment [37]. The ease of shaping potatoes into uniform cylindrical or rectangular phantoms akin to tumor dimensions makes them appealing for early-stage electroporation studies [34]. Their vegetable nature simplifies handling and eliminates ethical concerns and the need for Institutional Review Board (IRB) approval.

Biological tissues, such as potato phantoms, are anatomically and physiologically complex structures [20,38]. In addition, with their high water content, relatively low electrical resistance, uniform structure, and abundance and affordability, they are ideal for preclinical studies [39]. They are composed of individual living cells in an extracellular matrix [40]. The cell membrane consists of a thin (5 to 6 nm) nonconductive lipid bilayer. Table 4 shows their dielectric properties [41].

The dark color after 24 h of electroporation is due to the oxidation of phenolic compounds in the potato [42]. Electroporation disrupts the cell membrane, which allows phenolic compounds to encounter oxygen more easily. This can lead to a rapid browning of the potato tissue. The dark color after 24 h of electroporation is an indication of the distribution of the electrical field. The previous studies on potato electroporation have shown the effect of electrical pulses and the distribution of electric field [20,34,43] with different electrode arrangements. Our study has the potential to help future researchers to standardize the adherent cell electroporation experiments using the inset.

The use of insets, such as cell culture insets, permeable culture insets, plate insets, and tissue culture insets, is a common practice [44]. The insets are designed to maximize biological relevance and novel cell culture applications [45]. Our study shows, in both the potato experiments performed in 24- and 96-round-well plates and the FEA studies, that due to the inset, the electric field distribution is more uniform. Electroporation of the cell membrane occurs when a specific electric field strength threshold is overcome, and the transmembrane potential is able to permeabilize the membrane at some points [46].

The experiments performed to determine the electroporation threshold require a uniform electric field strength applied to the sample. The uniformity of the electric field application in the entire well bottom is crucial, since theelectroporation is a threshold phenomenon. Reversible electroporation occurs at a given electric field intensity range, and at electric field intensities that are too high, cells were irreversibly electroporated. Ivorra et al. [47] discussed that the best shape for a homogeneous electric field distribution inside the sample is that of plate electrodes fitting the culture chamber. The plated cells were an alternative to the use of cells in suspension. With suspension cells, the cell suspension is placed in a special cuvette that is equipped with plate electrodes with a fixed gap (1, 2, or 4 mm), which has the drawback that it is expensive and difficult to clean. Thus, using the insets, experiments with plated cells are handier and more repeatable.

The insets, which are easy to clean with alcohol solution or sodium hypochlorite and sterilized under UV, can be used to adapt the cylinder shape to a parallelepiped shape. Thus, this inset type might be used for classical 96-well plates (internal diameter 5 mm) with a reduced volume, where the inset with a plate electrode allows the application of uniform electric field strength. The reusability of the proposed solution could help reduce the plastic waste produced in laboratories and the experiment costs, making experiments more reproducible.

The proposed device might also be useful in 3D culture [48], as it can be used to test electroporation when the uniformity of the electric field requires stringent conditions. In this case, the samples are more expensive with respect to classical 2D culture, and the number of experiments used to test electroporation conditions had to be minimized.

Despite all their advantages, the use of insets, cells, and phantoms has certain limitations, such as the absence of critical biological attributes of cancer tissues, including extracellular matrix intricacies, vascularization, and the lack of essential cellular functions [49]. Hence, while potato phantoms are valuable for elucidating electroporation principles, they necessitate subsequent validation in more biologically relevant models.

## 5. Conclusions

A novel inset was designed and developed to obtain uniform electric field distribution in low-volume cell culture plates, such as the 24-well plates, and was tested using potato phantoms and HeLa cervical cancer cells. Our results indicate that using round well plates for electroporation experiments with the proposed novel inset design offers uniform electric field distribution, which contributes to effective electroporation results and reduces the experiment cost.

The inset is a practical device that might facilitate the uniform electroporation of cell cultures in classical round well culture plates. This device could enhance the experimental outcomes, since a cheaper classical culture plate can be used instead of a more expensive electroporation cuvette or Chamber Slides^TM^ System.

Potato tissue is an excellent representation of in vitro and in vivo samples for testing electroporation because it has a cellular and tissue structure similar to that of any live biological tissue. Its change of color to brown (dark) after a few hours is a good measure of the electroporation outcome.

The additional advantage of the presented inset design is that it can work for any size and shape of cell culture plates. Using the inset, the electrode gap remains constant, irrespective of the well type or electrode gap; hence, uniform fields could be achieved consistently. There is no need for any complex electrode geometry, and it can be used with simple everyday cell culture plates for electroporation research.

## Figures and Tables

**Table 1:** Dimensions of the 24- and the 96-well plates along with electrode thickness and gap.

Geometry	24—Well	96—Well
Diameter-D (mm)	16.20	7.15
Height-H (mm)	18.00	10.80
Width-W (mm)	10.00	3.30
Electrode thickness (mm)	1.00	0.10
Electrode gap (mm)	7.00	3.00

**Table 3:** Electroporation intensity analysis: average and median values in the analysis area in Figure 11.

	Background	Q1	Q2	Q3	Q4	Q5	L1s	L2s
24 well plate—average	50	97	77	70	--	--	85 ± 27	61 ± 12
24 well plate—median		88	61	55	--	--	80	58
Chamber slide—average	80	--	--	--	120	100	119 ± 35	115 ± 22
Chamber slide—median		--	--	--	114	96	107	111

**Table 4:** Dielectrical properties of potato tubers at 10 kHz and 1000 kHz.

Property	Frequency (kHz)	Potato
Capacitance (μF)	10	0.01628–0.0398
Capacitance (μF)	1000	0.001733–1.4165
Conductance (S)	10	0.0008167–0.03044
Conductance (S)	1000	0.0172–0.10153

## Data Availability

The original contributions presented in this study are included in the article. Further inquiries can be directed to the corresponding author.

## References

[1] Sahu P., Camarillo I.G., Sundararajan R. (2022). Enhanced Antiproliferation Potency of Electrical Pulse-Mediated Metformin and Cisplatin Combination Therapy on MDA-MB-231 Cells. Appl. Biochem. Biotechnol..

[2] Geboers B., Scheffer H.J., Graybill P.M., Ruarus A.H., Nieuwenhuizen S., Puijk R.S., van den Tol P.M., Davalos R.V., Rubinsky B., de Gruijl T.D. (2020). High-Voltage Electrical Pulses in Oncology: Irreversible Electroporation, Electrochemotherapy, Gene Electrotransfer, Electrofusion, and Electroimmunotherapy. Radiology.

[3] Wang L., Liu D., Zhou R., Wang Z., Cuschieri A. (2015). Tumour Cell Membrane Poration and Ablation by Pulsed Low-Intensity Electric Field with Carbon Nanotubes. Int. J. Mol. Sci..

[4] Yarmush M.L., Golberg A., Serša G., Kotnik T., Miklavčič D. (2014). Electroporation-Based Technologies for Medicine: Principles, Applications, and Challenges. Annu. Rev. Biomed. Eng..

[5] Review of Developments in Bioelectrics as an Application of Pulsed Power Technology. https://ieeexplore.ieee.org/document/10161581/.

[6] Martin R.C.G., McFarland K., Ellis S., Velanovich V. (2013). Irreversible Electroporation in Locally Advanced Pancreatic Cancer: Potential Improved Overall Survival. Ann. Surg. Oncol..

[7] Holland M.M., Bhutiani N., Kruse E.J., Weiss M.J., Christein J.D., White R.R., Huang K.-W., Martin R.C.G. (2019). A Prospective, Multi-Institution Assessment of Irreversible Electroporation for Treatment of Locally Advanced Pancreatic Adenocarcinoma: Initial Outcomes from the AHPBA Pancreatic Registry. HPB.

[8] Martin C.H., Martin R.C.G. (2023). Optimal Dosing and Patient Selection for Electrochemotherapy in Solid Abdominal Organ and Bone Tumors. Bioengineering.

[9] Corovic S., Lackovic I., Sustaric P., Sustar T., Rodic T., Miklavcic D. (2013). Modeling of Electric Field Distribution in Tissues during Electroporation. Biomed. Eng. Online.

[10] Golberg A., Bruinsma B.G., Uygun B.E., Yarmush M.L. (2015). Tissue Heterogeneity in Structure and Conductivity Contribute to Cell Survival during Irreversible Electroporation Ablation by “Electric Field Sinks”. Sci. Rep..

[11] Sieni E., Dettin M., Zamuner A., Conconi M.T., Bazzolo B., Balducci C., Di Barba P., Forzan M., Lamberti P., Mognaschi M.E. (2023). Finite Element Evaluation of the Electric Field Distribution in a Non-Homogeneous Environment. Bioengineering.

[12] Miklavcic D., Beravs K., Semrov D., Cemazar M., Demsar F., Sersa G. (1998). The Importance of Electric Field Distribution for Effective in Vivo Electroporation of Tissues. Biophys. J..

[13] Ivorra A., Al-Sakere B., Rubinsky B., Mir L.M. (2008). Use of Conductive Gels for Electric Field Homogenization Increases the Antitumor Efficacy of Electroporation Therapies. Phys. Med. Biol..

[14] Briko A., Kapravchuk V., Sorokina E., Baryshnikova M., Kosyrev V., Kosorukov V. Aspects of Effective Planning of Electroporation Procedure Using Finite Element Analysis Methods. Proceedings of the 2023 5th International Youth Conference on Radio Electronics, Electrical and Power Engineering (REEPE).

[15] Neal R.E., Garcia P.A., Robertson J.L., Davalos R.V. (2012). Experimental Characterization and Numerical Modeling of Tissue Electrical Conductivity during Pulsed Electric Fields for Irreversible Electroporation Treatment Planning. IEEE Trans. Biomed. Eng..

[16] Arena C.B., Szot C.S., Garcia P.A., Rylander M.N., Davalos R.V. (2012). A Three-Dimensional In Vitro Tumor Platform for Modeling Therapeutic Irreversible Electroporation. Biophys. J..

[17] Davalos R.V., Mir I.L.M., Rubinsky B. (2005). Tissue Ablation with Irreversible Electroporation. Ann. Biomed. Eng..

[18] Kranjc M., Miklavčič D., Miklavcic D. (2016). Electric Field Distribution and Electroporation Threshold. Handbook of Electroporation.

[19] Jeong S., Kim H., Park J., Kim K.W., Sim S.B., Chung J.H. (2021). Evaluation of Electroporated Area Using 2,3,5-Triphenyltetrazolium Chloride in a Potato Model. Sci. Rep..

[20] Boussetta N., Grimi N., Lebovka N.I., Vorobiev E. (2013). “Cold” Electroporation in Potato Tissue Induced by Pulsed Electric Field. J. Food Eng..

[21] Accelerate Motor, Sensor, and Actuator Design|Altair Flux. https://altair.com/flux.

[22] The Finite Element Method for Electromagnetic Modeling|Wiley. https://www.wiley.com/en-us/The+Finite+Element+Method+for+Electromagnetic+Modeling-p-9781848210301.

[23] Dirichlet Condition—An Overview|ScienceDirect Topics. https://www.sciencedirect.com/topics/mathematics/dirichlet-condition.

[24] Neumann Boundary Condition—An Overview|ScienceDirect Topics. https://www.sciencedirect.com/topics/engineering/neumann-boundary-condition.

[25] Filament Printer 3d MEDICAL Materials. https://www.smartmaterials3d.com/en/medical.

[26] Product Development. https://ultimaker.com/applications/product-development/.

[27] Leader in Biophysical Therapies|IGEA Medical. https://www.igeamedical.com/.

[28] Pavlin M., Kandušer M., Reberšek M., Pucihar G., Hart F.X., Magjarevićcacute R., Miklavčič D. (2005). Effect of Cell Electroporation on the Conductivity of a Cell Suspension. Biophys. J..

[29] Rols M.P., Golzio M., Gabriel B., Teissié J. (2002). Factors Controlling Electropermeabilisation of Cell Membranes. Technol. Cancer Res. Treat..

[30] Mir L.M., Glass L.F., Sersa G., Teissié J., Domenge C., Miklavcic D., Jaroszeski M.J., Orlowski S., Reintgen D.S., Rudolf Z. (1998). Effective Treatment of Cutaneous and Subcutaneous Malignant Tumours by Electrochemotherapy. Br. J. Cancer.

[31] Teissié J., Eynard N., Gabriel B., Rols M.P. (1999). Electropermeabilization of Cell Membranes. Adv. Drug Deliv. Rev..

[32] Lindelauf K.H.K., Thomas A., Baragona M., Jouni A., Nolte T., Pedersoli F., Pfeffer J., Baumann M., Maessen R.T.H., Ritter A. (2023). Plant-Based Model for the Visual Evaluation of Electroporated Area after Irreversible Electroporation and Its Comparison to in-Vivo Animal Data. Sci. Prog..

[33] Ivorra A., Mir L.M., Rubinsky B., Dössel O., Schlegel W.C. (2010). Electric Field Redistribution Due to Conductivity Changes during Tissue Electroporation: Experiments with a Simple Vegetal Model. IFMBE Proceedings, Proceedings of the World Congress on Medical Physics and Biomedical Engineering, Munich, Germany, 7–12 September 2009.

[34] Berkenbrock J.A., Brasil Pintarelli G., de Castro Antônio Júnior A., Suzuki D.O.H. (2019). Verification of Electroporation Models Using the Potato Tuber as In Vitro Simulation. J. Med. Biol. Eng..

[35] Berkenbrock J., Pintarelli G., Antonio A., Suzuki D. (2017). In Vitro Simulation of Electroporation Using Potato Model. CMBES Proc..

[36] Hjouj M., Rubinsky B. (2010). Magnetic Resonance Imaging Characteristics of Nonthermal Irreversible Electroporation in Vegetable Tissue. J. Membr. Biol..

[37] Castellví Q., Banús J., Ivorra A., Jarm T., Kramar P. (2016). 3D Assessment of Irreversible Electroporation Treatments in Vegetal Models. IFMBE Proceedings, Proceedings of the 1st World Congress on Electroporation and Pulsed Electric Fields in Biology, Medicine and Food & Environmental Technologies, Portorož, Slovenia, 6–10 September 2015.

[38] Janositz A., Noack A.-K., Knorr D. (2011). Pulsed Electric Fields and Their Impact on the Diffusion Characteristics of Potato Slices. LWT-Food Sci. Technol..

[39] Wardhana G., Raman N.M., Abayazid M., Fütterer J.J. (2022). Investigating the Effect of Electrode Orientation on Irreversible Electroporation with Experiment and Simulation. Int. J. CARS.

[40] Cima L.F., Mir L.M. (2004). Macroscopic Characterization of Cell Electroporation in Biological Tissue Based on Electrical Measurements. Appl. Phys. Lett..

[41] Soliman S.N., El-Sayed A.E. (2017). Dielectric properties of potato tubers related to storage conditions. Misr J. Agric. Eng..

[42] Akyol H., Riciputi Y., Capanoglu E., Caboni M.F., Verardo V. (2016). Phenolic Compounds in the Potato and Its Byproducts: An Overview. Int. J. Mol. Sci..

[43] Kranjc M., Bajd F., Serša I., de Boevere M., Miklavčič D. (2016). Electric Field Distribution in Relation to Cell Membrane Electroporation in Potato Tuber Tissue Studied by Magnetic Resonance Techniques. Innov. Food Sci. Emerg. Technol..

[44] Millicell® Cell Culture Inserts & Plates. https://www.sigmaaldrich.com/US/en/products/labware/cell-culture-and-cryogenics/millicell-cell-culture-inserts-and-plates.

[45] Millicell® Standing Cell Culture Inserts 0.4 Μm Pore Size Translucent Polycarbonate Membrane, 12 Mm Diam., 24-Well Standing|Sigma-Aldrich. http://www.sigmaaldrich.com/.

[46] Kotnik T., Kramar P., Pucihar G., Miklavcic D., Tarek M. (2012). Cell Membrane Electroporation- Part 1: The Phenomenon. IEEE Electr. Insul. Mag..

[47] Ivorra A., Villemejane J., Mir L. (2010). Electrical Modeling of the Influence of Medium Conductivity on Electroporation. Phys. Chem. Chem. Phys. PCCP.

[48] Zamuner A., Dettin M., Dall’Olmo L., Campana L.G., Mognaschi M.E., Conconi M.T., Sieni E. (2024). Development of 3D Melanoma Cultures on a Hyaluronic Acid-Based Scaffold with Synthetic Self-Assembling Peptides: Electroporation Enhancement. Bioelectrochemistry.

[49] Kim K.-H., An J., Park Y.-J., Park J.-H., Kim H.B., Yi J.-H., Kim H.-S. (2023). Tissue Ablation Using Irreversible Electrolytic Electroporation with Reduced Voltage. Electronics.

